# Varied Effects of Tending Ant Species on the Development of Facultatively Myrmecophilous Lycaenid Butterfly Larvae

**DOI:** 10.3390/insects10080234

**Published:** 2019-08-01

**Authors:** Takafumi Mizuno, Yasuo Hagiwara, Toshiharu Akino

**Affiliations:** 1 CAS Key Laboratory of Tropical Forest Ecology, Xishuangbanna Tropical Botanical Garden, Chinese Academy of Sciences, Menglun, Mengla 666303, China; 2 Applied Entomology Laboratory, Kyoto Institute of Technology, Saga-ippongi-cho 1, Kyoto 616-8354, Japan; 3 Faculty of Arts and Sciences at Fujiyoshida, Showa University, Fujiyoshida, Yamanashi 403-0005, Japan

**Keywords:** facultative myrmecophile, Lycaenidae, development, *Plebejus argyrognomon praeterinsularis*, species specific effect

## Abstract

Ants often tend and protect the larvae of various myrmecophilous lycaenid species, which influences the fitness of butterflies by altering their growth and developmental time. Tending produces diverse effects depending on lycaenid sex and the lycaenid/ant species combination. Effects are widely variable, especially in facultatively myrmecophilous lycaenids such as *Plebejus argyrognomon praeterinsularis*, because they are associated with several ant species and can survive without any ant tending. We studied the effects of ant tending on the adult body mass and larval developmental time of *P. argyrognomon praeterinsularis*. Female larvae grew significantly heavier as adults when tended by *Camponotus japonicus* rather than by either *Lasius japonicus* or no ant species. Ant tending did not affect the body mass of adult males or the developmental time of either male or female larvae. Thus, tending by *C. japonicus* could increase the fitness of *P. argyrognomon praeterinsularis* by increasing the mass of females without prolonging the duration of vulnerable immature stages, because larger females generally lay more eggs. This means that even facultatively myrmecophilous lycaenids might gain fitness benefits from particular ant species, which could be important in the conservation and management of at-risk species of facultatively myrmecophilous lycaenids.

## 1. Introduction

Generally, fitness is the combination of fecundity and survival. Fecundity in many insect taxa, including Lepidoptera, relates to body size [1,2], and in both sexes, larger mates are preferred over smaller ones, as large body size ensures greater mating success [3,4,5,6,7] and a higher number of eggs laid [8,9,10,11]. However, trade-offs between fecundity and larval survival are often found because increased larval developmental time or active foraging for growing large adults impose higher predation risk because of larval vulnerability to predators and parasitoids [12,13]. Thus, trade-offs between adult body size and larval development are closely related to fitness. Many lycaenid butterfly species have become myrmecophilous, by which they succeed in gaining fitness. Tending ants not only protect butterfly larvae from enemies, but they also affect larval body size and developmental time [14,15,16,17,18,19,20,21]; the influence of tending ants on the fitness of lycaenids can be substantial, although it varies considerably depending on the system.

Many lycaenid larvae have specialized organs to interact with ants: The so-called myrmecophilous organs. They provide nutritional rewards secreted from the dorsal nectary organ (DNO) to tending ants, and in return, they are protected from natural enemies by the ants [22,23,24]. They also have other myrmecophilous organs, such as tentacle organs (TOs), which had been thought to attract and excite tending ants by emitting volatile compounds to ward off enemies [23,24,25], but a recent study could not find any evidence for the secretory function of TOs in two species of Polyommatus lycaenid [26]. Pore cupola organs (PCOs) are single-celled epidermal glands that have been presumed to secrete substances capable of appeasing ants [24,27,28]. Such association between lycaenids and ants can be obligatory or facultative. In obligate myrmecophilous lycaenid species, the association with specific ant species is essential for survival, whereas facultatively myrmecophilous lycaenid species are associated with several ant species, but the associations are not essential for survival.

The tending of ants is possibly energetically costly for some lycaenid larvae because of the secretion of nutritional rewards, including sugars and amino acids [29,30,31]. It has been suggested that the production of the secretory substances negatively affects both adult mass and developmental time, which may ultimately reduce butterfly fitness [14,15,32]. However, several lycaenid species compensate or overcompensate for the energetic cost of the rewards [17,18,19,20,21,33,34], and such compensation is common in facultatively myrmecophilous lycaenids [18]. Moreover, it has been reported that the influence of tending ants on the body size and developmental time of lycaenids was different between sexes of lycaenids [16,18,19,21] and between tending ant species in facultatively myrmecophilous lycaenids [16,18,19,20].

*Plebejus argyrognomon praeterinsularis* is a facultatively myrmecophilous lycaenid, which is tended by several ant species in its natural habitat. This lycaenid is categorized as endangered (EN) on the Japanese Red List of 2018. They often pupate in ant nests, mainly in the nests of *Camponotus japonicus* (Hagiwara personal observation), and their pupal cuticular aldehydes suppress the aggressive behavior of *C. japonicus*, but do not suppress that of *Lasius japonicus* [35]. This suggests that *P. argyrognomon praeterinsularis* has a relatively closer relationship with *C. japonicus* than it does with other ant species. Accordingly, the lycaenids might gain greater benefits. For example, they may become larger adults, when they are tended by *C. japonicus* than by other ant species. However, if the primary benefit of ant association for this species is protection from predators, there could be no effect or even negative effects of tending on larval growth [14,15,32]. In this study, we investigated the influence of tending ants on the mass and developmental time of *P. argyrognomon praeterinsularis* under three ant-tending conditions: Rearing with *C. japonicus*, *L. japonicus*, and without any ant tending. We also investigated the difference in the amount of feces generated by lycaenid larvae and the frequency of nectar reward secretion and TO eversions among the ant-tending conditions to determine the mechanism of compensation for ant-related costs.

## 2. Materials and Methods

### 2.1. Capturing and Rearing of Study Insects

Mated female butterflies of *P. argyrognomon praeterinsularis* were collected in their natural habitat in Yamanashi Prefecture. They were individually kept in net cages (20 × 20 × 30 cm) with the host plant, *Indigofera pseudotinctoria*, to evoke oviposition. We released these butterflies in their original habitat after having collected eggs. Ten to 15 eggs collected from the host plant leaves were kept together in a plastic Petri dish (9-cm diameter) and incubated at 25 °C under a 14L: 10D photoperiod. Then, newly hatched larvae were moved to new Petri dishes and individually maintained with the host plant under the same temperature and photoperiod. No larvae had contact with any ant until the third instar stage, which was to standardize the experimental conditions. Foraging worker ants of *Camponotus japonicus* and *Lasius japonicus*, which have been reported to tend to *P. argyrognomon praeterinsularis* larvae and pupae in their natural habitat, were each collected from three colonies in the Kyoto Prefecture.

### 2.2. Lycaenid Rearing Experiment

We reared the third-instar larvae individually until adult eclosion in Petri dishes (9 cm diam. × 2-cm height) under three different ant-tending conditions, i.e., larvae were kept with two *C. japonicus* workers (Cj), larvae were kept with five *L. japonicus* workers (Lj), or larvae were kept without ants (noant). The experiment began just after the larvae molted into the third instar. We fed larvae with flowers and leaves of their host plant *I. pseudotinctoria*, replaced leaves and flowers with new ones every alternate day, and collected caterpillar feces at least every alternate day. We also fed the ants with 2 µL of sucrose solution (20%) twice, four or five days after beginning the experiment and two or three days after the lycaenid pupated. Such a small amount of sucrose would ensure ant survival during the experiment without preventing them from tending the lycaenids. The host plants were removed after the caterpillars pupated and pupae were reared under the same ant-tending condition as larvae until eclosion. As soon as the butterflies eclosed, they were moved to plastic cups (10-cm diameter × 9-cm height) and kept individually for a few hours for the passing of meconium. Next, the butterflies were killed in a deep freezer (−30 °C). The butterflies and collected caterpillar feces were placed in an incubator (50 °C) for 48 h to measure their dry mass. This experiment was conducted during the following three different periods: The middle of June, beginning of July, and beginning of August 2017. During the experiment, the number of worker ants tending larvae and pupae was recorded once to several times per day in each rearing Petri dish. We also recorded developmental time of third instar and fourth (final)-instar larvae and pupae; the mass of newly molted third instar and fourth instar larvae and pupae; the mass of dried adult butterflies, and dried feces. Larval mass gain was calculated by subtracting the mass of newly molted third-instar larvae from the mass of newly molted pupae. Pupal mass loss was calculated by subtracting the mass of newly molted pupae from the mass of dried adults, and relative pupal mass loss was also calculated by dividing pupal mass loss by mass of newly molted pupae.

To estimate the cost to the lycaenids in manipulating ants, we observed the behavior of third-instar and fourth-instar lycaenid larvae tended by both ant species for 5 min. The frequency of nectar reward secretion and eversion of TOs was recorded. We conducted the observation during daytime and avoided the period just after initiating the interaction between lycaenid and ants and after larvae had stopped feeding for molting or pupating.

### 2.3. Statistical Analysis

All the statistical analyses were conducted in R version 3.4.2. Linear mixed models (LMM) were used with the “lmer” function in the “lme4” package [36] for analyzing the mass of lycaenids at each developmental stage (third and fourth instar larva, pupa, and adult), mass gain during larval stages, pupal mass loss, relative pupal mass loss, dried feces mass, and developmental time of larvae and pupae. We set an ant-tending condition (categorical variable with three factors: Cj, Lj, and noant) and sex of the lycaenid (categorical variable with two factors, male and female) and interaction between these in the LMM analysis as fixed factors. Additionally, we set dried feces mass (continuous variable) as a fixed factor for the analysis of larval mass gain, and pupal mass (continuous variable) as a fixed factor for the analysis of pupal mass loss and pupal developmental time. For the analysis of relative pupal mass loss, we set pupal mass as an offset. The period when the experiments were conducted was set as a random effect in all the LMM analyses.

Generalized linear mixed models (GLMM) with a Poisson error distribution and log link function were used with the “glmer” function in the analysis of the frequency of nectar reward secretion and eversion of TOs. We set tending ant species (categorical variable with two factors, Cj and Lj), sex of lycaenids, and the interaction as fixed factors and individual lycaenids as a random factor. This GLMM analysis was conducted for each instar of lycaenid (third and fourth instar and pupa).

In LMM and GLMM analysis, we selected the best model based on corrected Akaike’s Information Criterion (AICc) and determined the statistical significance of each fixed factor in the best models by using a likelihood ratio test (LRT) with the ANOVA function. When the ant-tending condition or the interaction between it and the sex of the lycaenid were significant in the LMM analysis, we conducted multiple comparisons between ant-tending conditions using the “glht” function in the “multcomp” package [37].

We averaged the number of tending ants for each individual lycaenid in each instar (third and fourth instar larvae and pupa) and tested the significance of the difference between tending ant species and the sex of the lycaenid within each instar using the Steel-Dwass test.

## 3. Results

### 3.1. Mass of the Lycaenids in Each Developmental Stage

The best LMM models of body mass of pupae and dried adults were full models, which included the ant-tending condition, sex of the lycaenid, and the interaction between the two factors. The interaction terms significantly affected pupal mass (LRT, χ^2^ = 9.54, d.f. = 2, *p* = 0.00847) and dried adult mass (LRT, χ^2^ = 22.1, d.f. = 2, *p* < 0.001). Female pupae and adults tended by *C. japonicus* were significantly heavier than those under other ant-tending conditions. The mass of female pupae tended by *C. japonicus* was 8.48% and 13.4% greater on average than the mass of female pupa tended by *L. japonicus* and mass of female untended pupa, respectively (Cj versus Lj, *p* = 0.00895; Cj versus no-ant, *p* < 0.001; Lj versus no-ant, *p* = 0.634). Meanwhile, the mass of female adult tended by *C. japonicus* was 11.8% and 27.8% greater on average than the mass of female adults tended by *L. japonicus* and mass of female untended adults, respectively (Cj versus Lj, *p* < 0.001; Cj versus no-ant, *p* < 0.001; Lj versus no-ant, *p* = 0.0134) and the effect of tending ant species occurred only in females (Table 1). Whereas the difference in larval body mass between ant tending conditions and between sexes was not significant for newly shed third and fourth instar larvae (Table 1). The best LMM model of body mass of newly shed third instar larvae was the null model, reflecting an unbiased assignment of larvae to the ant tending conditions. The null model was also the best model for the mass of newly shed fourth instar larvae.

### 3.2. Mass Gain and Feces Mass during the Larval Stage

**Figure 1:**
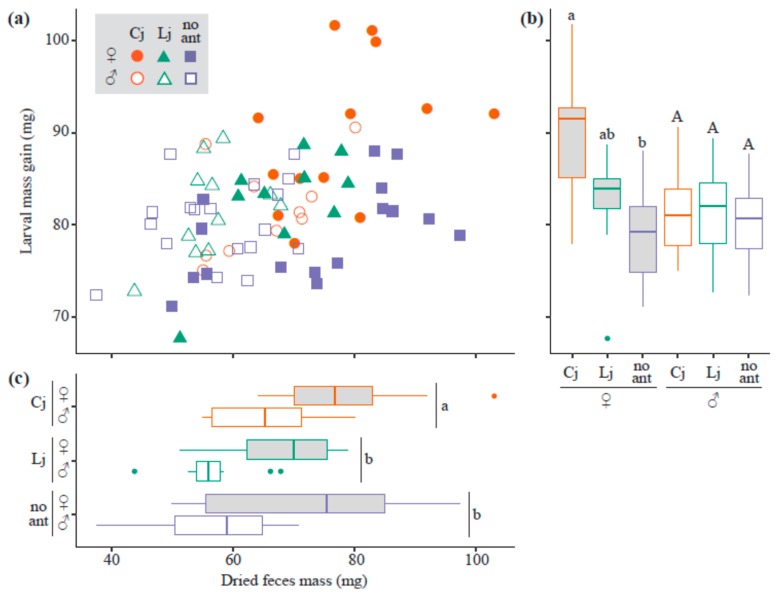
Larval mass gain and feces mass. A scatter plot shows the relationship between larval mass gain and dried feces mass (**a**). Orange circle, green triangle, and blue square symbols represent larvae tended by *C. japonicus* (Cj), by *L. japonicus* (Lj), and untended larvae (no ant), respectively. Solid and open symbols represent female and male larvae, respectively. Box plots show larval mass gain (**b**) and dried feces mass (**c**) between ant-tending conditions (Cj: orange, Lj: green, no ant: blue) and lycaenid sex (female: gray boxes, male: white boxes). Different letters represent significant differences.

The full model explaining larval mass gain (i.e., including ant-tending condition, sex of lycaenids, the interaction between these two factors, and dried feces mass as explanatory variables) accounted for the most variation among trials. Feces mass and the interaction significantly affected larval mass gain (LRT, χ^2^ = 16.5, d.f. = 1, *p* < 0.001 and χ^2^ = 10.2, d.f. = 2, *p* = 0.00617, respectively). Larval mass gain was highly correlated with dried feces mass (Figure 1a), and was affected by the ant-tending conditions when larvae were female. Female larval mass gain was 13.5% greater in larvae tended by *C. japonicus* than in untended female larvae, and the difference was significant (*p* < 0.00, Figure 1b). Dried feces mass was significantly affected by ant-tending conditions (LRT, χ^2^ = 16.6, d.f. = 2, *p* < 0.001) and the sex of the lycaenid (LRT, χ^2^ = 27.9, d.f. = 1, *p* < 0.001), but not by their interaction. Feces mass was 24.1% greater in females than in males and 16.4% and 10.9% greater in larvae tended by *C japonicus* than in larvae tended by *L. japonicus* and untended larvae respectively (Cj versus Lj and Cj versus no-ant, *p* < 0.001; Lj versus no-ant, *p* = 0.932; Figure 1c).

### 3.3. Mass Loss from Pupae to Adults

**Figure 2:**
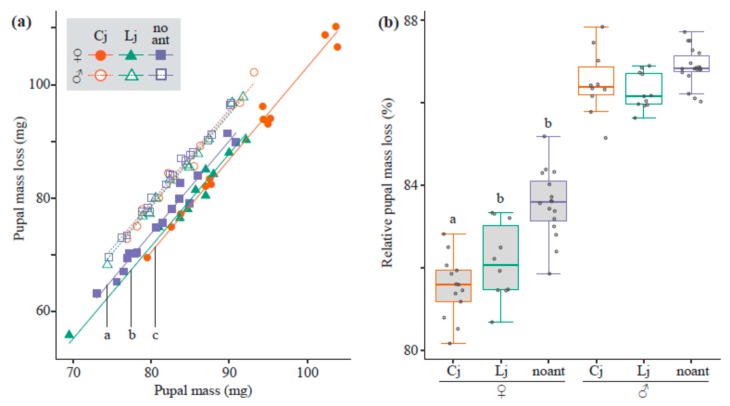
Pupal mass loss and relative pupal mass loss. The plot shows the relationship between pupal mass loss and pupal mass (**a**). Different shapes, such as points, orange circles, green triangles, and blue squares, and different line types, including sold, dotted, and dashed, represent different ant-tending conditions; larvae tended by *C. japonicus* (Cj), by *L. japonicus* (Lj), and untended larvae (no ant), respectively. Solid and open symbols represent female and male larvae, respectively. The boxplot shows relative pupal mass loss among ant tending conditions (Cj: orange, Lj: green, no ant: blue) and between sexes of lycaenids (female: gray boxes, male: white boxes) (**b**). All the data points are shown as dots. Dots outside boxes and whiskers are outliers. In both graphs, different letters represent significant differences.

The full model explaining pupal mass loss (i.e., including the ant-tending conditions, sex of the lycaenid, interaction between these two, and pupal mass as explanatory variables) accounted for the most variation among trials. Pupal mass and the interaction significantly affected pupal mass loss (LRT, χ^2^ = 334.2, d.f. = 1, *p* < 0.001 and χ^2^ = 18.6, d.f. = 2, *p* < 0.001, respectively). Pupal mass loss was highly correlated with pupal mass (Figure 2a) and was on average 1.98% greater in males (72.1 mg ± 0.661, mean ± standard error) than in females (70.7 mg ± 0.970). Ant-tending conditions affected this in females only. In females, the average mass loss of pupae tended by *C. japonicus* (75.1 mg ± 1.83) was 7.75% and 10.6% greater than those of pupae tended by *L. japonicus* (69.7 mg ± 1.53) and those of untended pupae (67.9 mg ± 1.06), respectively. However, after considering pupal mass, tending by *C. japonicus* significantly decreased pupal mass loss to a greater extent than did tending by *L. japonicus* and the untended condition (Cj versus Lj, *p* = 0.0375; Cj versus no-ant, *p* < 0.001), and tending by *L. japonicus* significantly decreased pupal mass loss more than did the untended condition (Lj versus no-ant, *p* < 0.001). Relative pupal mass loss clearly showed decreasing pupal mass loss when considering pupal mass. The full model of relative pupal mass loss that included ant-tending condition, sex of lycaenid, and the interaction as explanatory variables was the best model, and the interaction significantly affected relative pupal mass loss (LRT, χ^2^ = 6.71, d.f. = 2, *p* = 0.0348). Relative pupal mass loss in females (82.5% ± 0.195) was lower than in males (86.6% ± 0.0957), and the ant-tending condition affected only female lycaenids (Figure 2b). The relative pupal mass loss of females tended by *C. japonicus* (81.5% ± 0.208) was significantly lower than that in females tended by *L. japonicus* (82.2% ± 0.290; Cj versus Lj, *p* < 0.001) and untended female (83.6% ± 0.206; Cj versus no-ant, *p* < 0.001), but there was no difference between that of females tended by *L. japonicus* and untended females (Lj versus no-ant, *p* = 0.848).

### 3.4. Developmental Time for Larvae and Pupae

**Figure 3:**
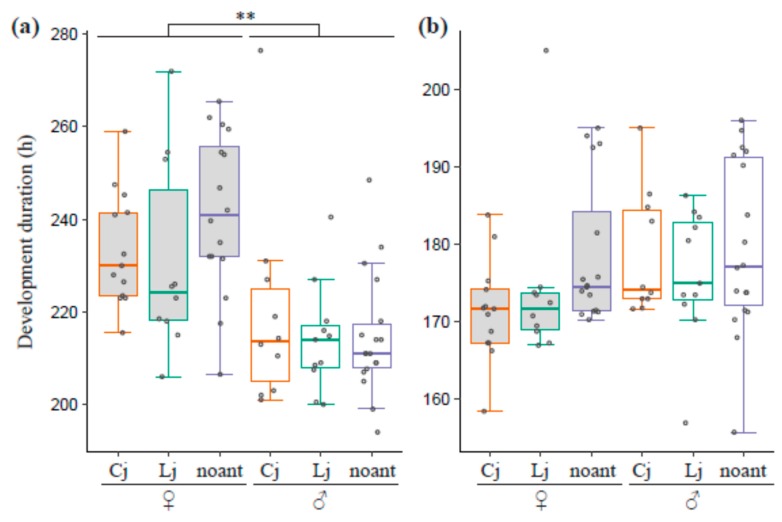
Developmental time of the larval and pupal stage. The boxplots show the developmental time from the third instar to pupation (**a**) and from pupation to eclosion (**b**). The colors of the box outlines and the dots represent ant-tending conditions (Cj: orange, Lj: green, no ant: blue). All the data points are shown as dots. Dots outside boxes and whiskers are outliers. The gray and white boxes represent female and male lycaenids, respectively. ** indicates a significant difference between sexes.

The best model for larval developmental time included only one fixed factor: the sex of lycaenids. The developmental time of female lycaenid larvae was 9.41% (about 20 h) longer on average than that of males, and the difference was significant (LRT, χ^2^ = 25.5, d.f. = 1, *p* < 0.001, Figure 3a). In the case of pupal developmental time, the best model only had the sex of the lycaenid as a fixed factor, but this was not statistically significant (LRT, χ^2^ = 2.37, d.f. = 1, *p* = 0.123, Figure 3b).

### 3.5. Frequency of Secreting Reward and TO Eversion

**Figure 4:**
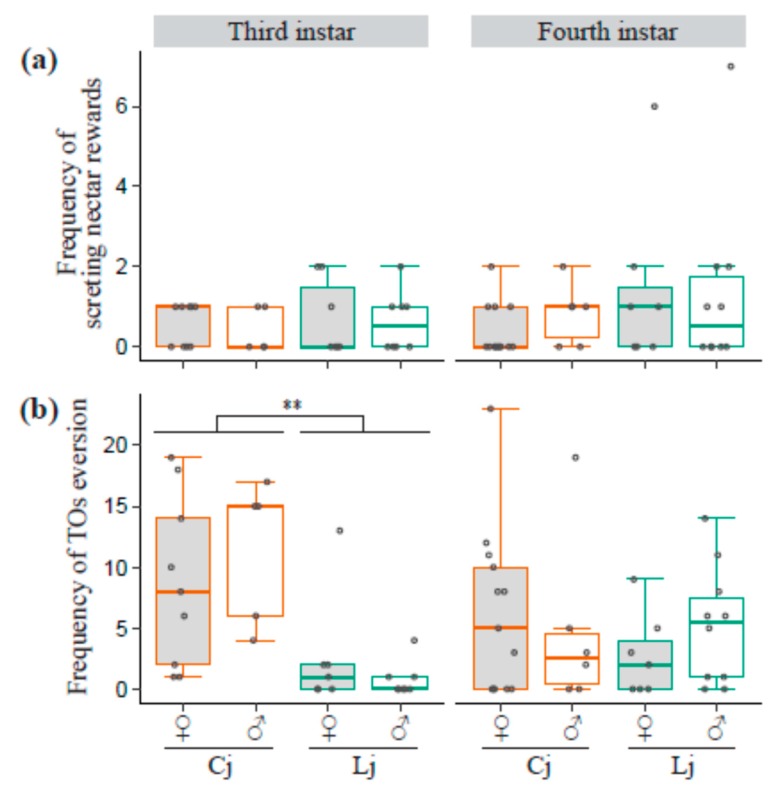
Frequency of nectar reward secretion and tentacle organs (TO) eversion. The boxplots show the frequency of nectar reward secretion (**a**) and TO eversion (**b**). The colors of the box outlines and the dots represent ant-tending conditions (Cj: orange, Lj: green). All the data points are shown as dots. Dots outside boxes and whiskers are outliers. The gray and white boxes represent female and male lycaenid larvae, respectively. ** indicates a significant difference between tending ant species.

All the fixed factors, including the ant-tending condition, sex of lycaenid, and the interaction of the two did not significantly affect the frequency of nectar reward secretion. The null model was the best model in the third and fourth instar larvae (Figure 4a). The third instar larvae tended by *C. japonicus* significantly produced TO eversions more frequently than those tended by *L. japonicus* did (LRT, χ^2^ = 18.9, d.f. = 1, *p* < 0.001, Figure 4b). Whereas in the fourth instar larvae, no fixed factors had significant effects on the frequency of TO eversions (Figure 4b).

### 3.6. Number of Tending Ants

**Figure 5:**
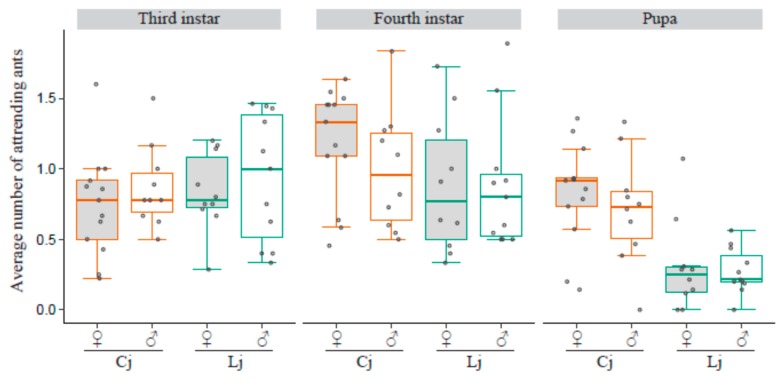
Number of tending ants for lycaenid larvae and pupae. The boxplots show the average number of tending ants for female (gray box) and male (white box) lycaenids in each developmental stage, third instar larvae, fourth instar larvae, and pupae. The colors of the box outlines and the dots represent ant-tending conditions (Cj: orange, Lj: green). All the data points are shown as dots. Dots outside boxes and whiskers are outliers. Different letters represent significant differences.

The number of tending ants was not significantly different between sexes of lycaenids and tending ant species in terms of the third and fourth instar larvae. Although *C. japonicus* more frequently tended to pupae than *L. japonicus* did (females tended by Cj versus females tended by Lj *p* = 0.0350, males tended by Cj versus males tended by Lj *p* = 0.0248, females tended by Cj versus males tended by Lj *p* = 0.00777), the number of *C. japonicus* tending to male pupae was not significantly different from that of *L. japonicus* tending to female pupae (*p* = 0.0875, Figure 5).

## 4. Discussion

Myrmecophilous lycaenid larvae secrete nutritious rewards for ant guards, which imposes a cost on the lycaenid, sometimes resulting in decreased adult mass or prolonging duration of the immature life stage [14,15,32]. However, *P. argyrognomon praeterinsularis* overcompensated in females and compensated in males for the cost of ant tending. Ant tending neither decreased the adult mass nor increased larval and pupal developmental time. Moreover, *C. japonicus* increased larval mass gain and decreased relative pupal mass loss in females, resulting in female larvae tended by *C. japonicus* growing into heavier adults. This result strongly suggests that *C. japonicus* increases the reproductive fitness of the lycaenid, because heavier female butterflies of *P. argyrognomon praeterinsularis* can probably lay more eggs, as demonstrated in several lepidopterans [8,9,10,11]. Feces mass increased when larvae were tended by *C. japonicus*, but the frequency of nectar reward secretion was not significantly different between the sexes of lycaenids or between ant species, which suggested that part of the mechanism of compensation is an enhancement in food intake. These results indicate that *P. argyrognomon praeterinsularis* adapts to the association with *C. japonicus* better than to that with *L. japonicus*. Our results are also supported by observations that the lycaenids often pupated in *C. japonicus* nests (Hagiwara, personal observation) and by our previous study, wherein it was shown that the pupal cuticular chemicals of the lycaenids suppressed the aggressiveness of *C. japonicus* but not of *L. japonicus* [35].

In the larval stage, ant-tending conditions significantly affected mass gain only in the female lycaenid. Mass gain of larvae tended by *C. japonicus* was the greatest, followed by that of larvae tended by *L. japonicus*, and that of untended larvae. One possible mechanism for these results is an increased larval food intake, which is supported by our results wherein the feces mass of larvae tended by *C. japonicus* was greater. Ant tending can increase time for feeding by varying the larval feeding behavior, even though these results showed that ant tending did not prolong the larval stage. If larvae tended by ants keep feeding and untended larvae stop feeding when disturbed by potential predators, larvae tended by ants have more time to feed than do untended larvae. Under rearing conditions, replacing host plants and cleaning rearing cases can serve as disturbances. The foraging patterns adopted by the larvae of different lycaenid species depends in part on the traits of the tending ant species, their activity patterns, and the quality of protection [38]. For example, in the case of ants that are assiduous tenders, are diurnally active, and possess a large colony size and/or a well-developed mass recruitment system, the lycaenid larvae might feed on the terminal foliage during the day time where leaves are more nutritious, despite increased visibility to predators such as *Jalmenus evagoras* [38]. Whereas in the case of ants that are relatively weak tenders, are nocturnal or crepuscular foragers, and possess small colony sizes and/or poor means of mass recruitment, the lycaenid larvae might resort to a more cryptic mode of foraging such as *Ogyris genoveva* and *Polyommatus bellargus* [38,39]. Such foraging patterns are likely inherent. While it was not reported that larvae can change their feeding behavior in response to the presence or absence of ants, or to tending ant species in previous studies on lycaenids [32,40,41], it was reported in Riodinidae *Eurybia elvina* larvae [42]. When tended by *C. japonicus*, mass gain of the lycaenids increased only in females, whereas feces mass increased in both sexes, which suggested the existence of a mechanism for increased mass gain due to ant tending that is not based on increased food intake. Another possible mechanism that is compatible with food intake enhancement is that ant tending may affect assimilation efficiency. Baylis and Pierce [32] and Wagner and Rio [40] tested the influence of ant tending on the assimilation efficiency of obligatory and facultatively myrmecophilous lycaenid larvae, but found no evidence that the ants had enhanced assimilation efficiency. However, some lepidopteran larvae are able to plastically alter their assimilation efficiency as reported for *Manduca sexta*, which alter their assimilation efficiency in response to predation risk [43].

Female-biased mass gain influenced by ant tending might be related to the differential benefit based on the large body size of males relative to that of females. Some studies suggested that having a large size is related to the high mortality rate, because predators prefer larger prey [44], but see [45]. Greater food-searching activity, which is needed in order to grow large, is also related to the high mortality rate, because prey with greater activity are found easily by predators [12,13]. Lepidopteran larvae normally do not grow at their maximum growth rate, which is thanks in part to predation risk to large body size [46,47]. This constrained growth rate may be released when the larvae are tended by ants, since ant tending can be an indicator of lower predation risk. Particularly in females, the benefit of being larger is likely to outweigh the potential cost, because female body size is highly related to egg production (i.e., reproductive fitness) [8,9,10,11]. Additionally, not only body mass but also ant tending itself might affect fecundity. In *J. evagoras*, although ant tending decreased final adult size, fecundity was greater in tended females than in untended same-sized counterparts [48]. On the contrary, a large body size may not provide a benefit to male adults in this lycaenid species, since ant tending did not increase male mass. However, the feces mass of male larvae tended by *C japonicus* increased, suggesting that *C. japonicus* might change the physiology of males. Thus, the effect of ant tending on the development of immature lycaenid greatly varies depending on the lycaenid sex [16,18,19,20,34]. This is probably related to reproductive strategy; for example, *Polyommatus icarus* males get larger than females under ant-tending conditions [16], which is probably because of the importance of male–male competition for access to females [49]. The effect of ant tending also varies depending on the tending ant species [16,18,19,20,34], which might be correlated with the defensive efficiency of each ant species. However, there are few studies regarding the difference in actual defensive efficiency between tending ant species [50].

Mass loss and relative mass loss were greater in males than in females, and ant tending decreased these parameters in females only, which is another cause for the differences in adult mass. Female Lepidopterans typically lose less water upon eclosion due to the water allocation to eggs, than do their conspecific males, which is a primary cause for the difference in mass loss between sexes [51]. In this study, we measured dried adult mass, and the difference in adult water content between sexes did not reflect adult mass. If the relative water content of pupae were the same between sexes and independent of ant-tending conditions, larger pupae would have lost more mass, because they would have greater water content when compared with smaller pupae. However, the mass loss and relative mass loss in females was less than those in males despite the greater pupal mass of females, and relative mass loss in female pupae tended by *C. japonicus* was less than that of female pupae under other conditions, despite the greater mass in pupae tended by *C. japonicus*. These results suggest that larger pupae, especially females tended by *C. japonicus*, retain more substance for allocation to their adult forms.

*P. argyrognomon praeterinsularis* likely benefits more from associating with *C. japonicus* than with *L. japonicus*. Although the lycaenid larvae apparently need to secret more nectar reward to be tended by *C. japonicus* than by *L. japonicus* because the body size of *C. japonicus* (around 10 mm) is larger than that of *L. japonicus* (around 3 mm), the frequency of secreting the nectar reward, which is costly for lycaenid larvae [14,15,32], was not significantly different between the two ant-tending conditions. However, the frequency of TO eversion, which was probably not very costly for lycaenid larvae [23], was significantly higher only in the third instar larvae tended by *C. japonicus*. TO eversion, which enables ants deter enemies, is thought to be a defensive behavior [23,24,25], and we observed *C. japonicus* responding sensitively to TO eversion and exhibiting charging behavior, while *L. japonicus* was not as sensitive. Moreover, we found more *C. japonicus* workers tending the lycaenid pupae, when compared with the tending behavior of *L. japonicus*, despite fewer *C. japonicus* workers (two individuals) being placed in each rearing Petri dish in comparison with *L. japonicus* workers (five individuals). Given the difference in the number of ant workers for each ant species, although the average number of ants that tended larvae was not significantly different between *C. japonicus* and *L. japonicus*, the rate of ants tending larvae and pupae was higher in *C. japonicus* than in *L. japonicus*. This suggests that *P. argyrognomon praeterinsularis* elicits tending behavior more effectively in *C. japonicus* than in *L. japonicus*.

The ant workers that were used for the experiments were isolated from their colonies and kept under lab conditions during the experiment. It is possible that their behavior is different in the lab environment than in their natural condition. However, the tending ants of both species were actively palpating larvae and pupae with their antennae, received nectar rewards, and showed charging behavior in response to TO eversion; this was especially noted in *C. japonicas.* These behaviors are also shown by tending ants under natural conditions [24]. Thus, we assume that the influence of tending ants on the lycaenids in our experiment was almost the same as that under natural conditions.

## 5. Conclusions

Female larvae of *P. argyrognomon praeterinsularis* became larger adults when tended by *C. japonicus* than by *L. japonicus* or left untended, without having a prolongation of their developmental time. A part of the mechanism is that the food intake of larvae tended by *C. japonicus* was enhanced, resulting in increased feces mass. Ant tending generally decreased predation risk; the pupae of *P. argyrognomon praeterinsularis* were tended by *C. japonicus* more easily than by *L. japonicus*. Additionally, *P. argyrognomon praeterinsularis* sometimes pupated in the nest of *C. japonicus* and pupal cuticular aldehydes could appease only *C. japonicas* [35]. This evidence suggests that *P. argyrognomon praeterinsularis* is more closely associated with *C. japonicus* than with *L. japonicus*, and the fitness of *P. argyrognomon praeterinsularis* increases more when *C. japonicus* tends to them. Thus, the presence of *C. japonicus* might be important for the conservation of this endangered lycaenid species. The influence of the invasive plant *Eragrostis curvula* on the host plant of this lycaenid and the butterfly’s conservation thereof has been evaluated previously [52]. However, to date, the influence of tending ant species has not been reported. Therefore, future studies need to investigate whether the effects of ants, particularly *C. japonicus*, on the lycaenid, as demonstrated in the current study, are observed under field conditions, the extent to which ant presence impacts predation, and the importance such effects play in their population.

## Figures and Tables

**Table 1:** Mass of newly shed larvae, pupae, and dried adults.

Ant Tending Condition	Sex	Average Mass ± Standard Error (mg)				n
		3rd	4th	Pupa	Adult	
Cj	f	2.3 ± 0.1	15.1 ± 0.6	92.1 ± 2.3 a	17.0 ± 0.5 a	13
Lj	f	2.3 ± 0.2	13.6 ± 0.7	84.9 ± 2.0 b	15.2 ± 0.5 b	10
no-ant	f	2.1 ± 0.1	15.3 ± 0.4	81.2 ± 1.3 b	13.3 ± 0.3 c	16
Cj	m	2.0 ± 0.1	13.6 ± 1.0	83.7 ± 1.7 A	11.3 ± 0.3 A	10
Lj	m	2.1 ± 0.1	14.7 ± 0.9	83.7 ± 1.6 A	11.5 ± 0.3 A	11
no-ant	m	2.3 ± 0.1	14.4 ± 0.7	82.6 ± 1.1 A	10.9 ± 0.2 A	17

Cj, Lj, and no-ant represent the lycaenids that were reared with *Camponotus japonicus*, *Lasius japonicus*, or without ants, respectively. Different letters behind standard errors represent significant differences between ant tending condition within pupal and adult stages. Lowercase and uppercase letters are for females and males, respectively.
